# *Ednrb* ^*−/−*^ mice with hirschsprung disease are missing Gad2-expressing enteric neurons in the ganglionated small intestine

**DOI:** 10.3389/fcell.2022.917243

**Published:** 2022-07-26

**Authors:** Sukhada Bhave, Richard A. Guyer, Nicole Picard, Meredith Omer, Ryo Hotta, Allan M. Goldstein

**Affiliations:** Department of Pediatric Surgery, Massachusetts General Hospital, Harvard Medical School, Boston, MA, United States

**Keywords:** hirschsprung disease, enteric nervous system, enteric neurons, endothelin receptor type B, single-cell RNA sequencing

## Abstract

Hirschsprung disease is most often characterized by aganglionosis limited to the distal colon and rectum, and mice lacking the Endothelin receptor type B (Ednrb) faithfully recapitulate this phenotype. However, despite the presence of enteric ganglia in the small intestine, both human patients and Ednrb−/− mice suffer from dysmotility and altered gastrointestinal function, thus raising the possibility of enteric nervous system (ENS) abnormalities proximal to the aganglionic region. We undertook the present study to determine whether abnormalities with the ENS in ganglionated regions may account for abnormal gastrointestinal function. We performed single-cell RNA sequencing on ENS cells from the small intestine of Ednrb−/− mice and compared the results to a published single-cell dataset. Our results identified a missing population of neurons marked by the enzyme Gad2, which catalyzes the production of *γ*-Aminobutyric acid (GABA), in the small intestine of Ednrb−/− animals. This result was confirmed by immunostaining enteric ganglia from Ednrb−/− mice and their wild-type littermates. These data show for the first time that ganglionated regions of the Hirschsprung gut lack a neuronal subpopulation, which may explain the persistent gastrointestinal dysfunction after surgical correction of Hirschsprung disease.

## Introduction

The enteric nervous system (ENS), a complex network of neurons and glia organized into ganglia along the gastrointestinal (GI) tract, is essential in regulating gut motility (Furness, 2012; Goldstein et al., 2013; Spencer and Hu, 2020). Enteric neuropathies arise from either congenital absence or postnatal injury or loss of the ENS, and result in significant morbidity. Hirschsprung disease (HSCR) is an enteric neuropathy characterized by congenital absence of ganglion cells along a variable length of distal intestine due to failure of neural crest cells to complete their colonization of the entire intestine during development (Gershon and Ratcliffe, 2004; McKeown et al., 2013), causing the distal aganglionic intestine to be functionally obstructed. The current treatment for HSCR involves surgical resection of the aganglionic intestine. While surgery is lifesaving, HSCR patients often suffer from lifelong gastrointestinal dysfunction, including constipation, fecal incontinence, and enterocolitis, which significantly reduces their quality of life (Yanchar and Soucy, 1999; Miele et al., 2000; Tomita et al., 2003).

Persistent bowel dysfunction in HSCR patients after pullthrough surgery implies the presence of diffuse abnormalities in the intestine that are not limited to the aganglionic distal segment. Research on patients with HSCR and in animal models has suggested that the ganglionic intestine may not be normally innervated. This is supported by aberrant ratios of neurochemical markers in the ganglionated proximal colon. Specifically, an overabundance of nitrergic innervation and a deficit of cholinergic innervation has been reported in the proximal colon of HSCR patient samples and mice with HSCR (Zaitoun et al., 2013; Cheng et al., 2016). Furthermore, several animal models of HSCR, including *Ednrb* ^*−/−*^, *Et-3* ^*−/−*^, and *Gdnrf* ^*+/-*^ mice, which are characterized by variable lengths of aganglionic rectum and distal colon (Ro et al., 2006; Barnes and Spencer, 2015), exhibit a hypoganglionic phenotype in the colon proximal to the aganglionic segment characterized by decreased myenteric neuronal density (Roberts et al., 2008; Bhave et al., 2021). These changes in the proximal colon are associated with altered motility (Ro et al., 2006). We recently demonstrated that these structural ENS abnormalities in HSCR extend proximally to the small intestine and stomach. *Ednrb* ^*−/−*^ mice showed reduced density of myenteric ganglia and neuronal fibers in the small intestine while the myenteric neuronal packing density was increased in the stomach (Bhave et al., 2021).

We undertook the present study to identify ENS abnormalities in the ganglionated small intestine of the *Ednrb* ^*−/−*^ mouse model of HSCR using single-cell RNA sequencing (scRNA-seq). Understanding the deficits in the ganglionated ENS of the proximal intestine in HSCR will have significant implications for predicting post-surgical outcome and guiding treatment strategies in patients with post pull-through bowel dysfunction.

## Materials and methods

### Animals

This study was conducted in accordance with the protocols reviewed and approved by the Institutional Animal Care and Use Committee at Massachusetts General Hospital. The following mice were obtained from Jackson Laboratory (Bar Harbor, ME, United States): *Ednrb* ^*+/-*^ mice (B6; 129-Ednrb^tm1Ywa^/J, stock #003295), *Wnt1* ^*Cre/+*^ mice (B6. Cg-Tg (Wnt1-Cre)11*R*th/MileJ, stock #009107), and *GCaMP5* ^*tdT/+*^ mice (B6; 129S6-Polr2a^Tn(pb−CAG-GCaMP5g,−tdTomato)Tvrd^/J, stock #024477). We crossed *Wnt1::Cre; Ednrb* ^*+/-*^ and *GCaMP5-tdT; Ednrb* ^*+/-*^ mice to obtain *Wnt1-tdT; Ednrb* ^*+/+*^ (*Ednrb-*wildtype) and *Wnt1-tdT; Ednrb* ^*−/−*^ mice. *Ednrb* ^*−/−*^ homozygous pups were identified by a white coat color and exhibit distal aganglionosis Mice were genotyped using polymerase chain reaction to distinguish between *Ednrb* ^*+/+*^ and *Ednrb* ^*−/−*^ littermates (Thiagarajah et al., 2014).

### Isolation of ENS cells

Postnatal day 14 *Wnt1-tdT; Ednrb* ^*−/−*^ mice (*n* = 3) were euthanized and their small intestines were dissected and placed in ice-cold PBS. The longitudinal muscle myenteric plexus (LMMP) layer, which contains myenteric ganglia, was separated from the underlying submucosa using fine forceps under a dissecting microscope. After dissection, LMMP tissue was enzymatically digested with dispase (250 μg/ml; StemCell Technologies, Vancouver, BC) and collagenase XI (1 mg/ml; Sigma Aldrich, St. Louis, MO) at 37°C for 40 min. Single cells were isolated by filtration through a 40 μm filter. tdTomato-positive cells were collected by fluorescence activated cell sorting (FACS) using a BD Bio-sciences FACSAria sorting instrument.

### Preparation of single-cell RNA-sequencing libraries

LMMP tissue was dissected from the entire small intestine (duodenum to terminal ileum) of three separate *Wnt1-tdT; Ednrb* ^*−/−*^ mice and sorted for tdTomato + cells as described above. Immediately after sorting, cells were manually counted with Trypan blue to assess viability. After determining viable cell counts, a 10X Chromium Controller located in our facility was used along with 10X Genetics (Pleasanton, CA) v3.1 kits to generate gel bead emulsions (GEMs), followed by library preparation according to the manufacturer’s protocol.

### Sequencing and genome alignment

Sequencing was performed at the Harvard University Bauer Core Facility, where libraries were sequenced on either Illumina NextSeq or Illumina NovaSeq instruments. Demultiplexing, genome alignment, and feature-barcode matrix generation was performed with the 10X Genetics Cell Ranger software pipeline (Zheng et al., 2017).

### Single-cell data analysis

ScRNA-seq data was analyzed with the open-source Seurat package implemented in the R computing environment^4^. All three datasets were filtered for cells containing between 1,500 and 5,000 unique genes per cell, and <12.5% mitochondrial genes. After filtering, datasets were merged, and potentially confounding genes were removed, as previously described (Morarach et al., 2021). Neighbors were identified and UMAP projection was performed using the first 20 principal components. Clusters were identified using the “FindClusters” command with resolution = 0.5 using the Louvain algorithm (Blondel et al., 2008).

### Published scRNA-seq data

ScRNA-seq of the normal mouse ENS at P21 was published previously (Morarach et al., 2021). This data was obtained from the NCBI Gene Expression Omnibus, accession GSE149524. Analysis of this data was performed with Seurat in the R environment. Briefly, the datasets were filtered for cells with between 1,500 and 8,000 unique genes detected, and <12.5% mitochondrial genes. Datasets were then integrated using the standard Seurat workflow. Following integration, PCA was performed and UMAP projection was undertaken with the top 30 principle components, and clusters were identified using the “FindClusters” command with resolution = 0.5 using the Louvain algorithm (Blondel et al., 2008).

### Tissue preparation and immunostaining

Tissue preparation and immunohistochemistry were performed as previously described^7^. Whole mount LMMP preparations were obtained from the small intestines of 2–3 weeks old *Ednrb* ^*−/−*^ mice and wildtype littermates. LMMP preparations were fixed with 4% PFA for 6 h and blocked with 10% donkey serum/10% bovine serum albumin/1% triton in PBS for 1 h at room temperature. Samples were incubated overnight at 4°C in primary antibodies, which were diluted in blocking buffer. Primary antibodies included human anti-HuC/D (Anna-1, 1:10,000, kindly gifted by Lennon lab), rabbit anti-MATH2/NEUROD6 (5 μg/ml, Abcam ab85824), rabbit anti-GAD65/GAD2 (1:100, Invitrogen PA5-22260), rabbit anti-nNOS (1:400, Invitrogen 61-7000), and rabbit anti-Calretinin (1:200, Invitrogen 18-0211). The preparations were washed in PBS prior to adding the secondary antibodies, which were diluted in blocking buffer. The samples were incubated for 3 h at room temperature in the secondary antibodies. These antibodies included donkey anti-rabbit IgG (1:500; Alexa Fluor 546; Invitrogen A10040) and donkey anti-human IgG (1:200, Alexa Fluor 488; Jackson ImmunoResearch Laboratories 709-545-149). Cell nuclei were stained with DAPI (Invitrogen D1306). Samples were mounted with aqua-poly/mount (Polysciences Inc. 18,606-20).

### Image acquisition and analysis

High-power images of the myenteric ganglia were taken using Keyence BZX-700 All-In-One Microscopy (Keyence America Itasca, IL). Images were processed using ImageJ software (National Institutes of Health, Bethesda, MD). Neuronal subtypes in wild-type and KO mice (*n* = 3-4 mice per group) were quantified using wholemount LMMP preparations of the proximal small intestine (5 cm segment of duodenum) and distal small intestine (5 cm segment of distal ileum). High-power z-stack images of the LMMP preparations were taken and analyzed in ImageJ. Hu + neuronal cell bodies were analyzed per high-power field from 2-3 images per tissue and the percentage of Hu + neurons expressing Gad2, Neurod6, nNOS, and Calretinin, were calculated. Statistical analysis was performed using Prism 8 (GraphPad software, Inc., La Jolla, CA, United States). Statistical significance between wild-type and *Ednrb* ^*−/−*^ mice was assessed using Student’s t-test. with *p*-values < 0.05 regarded as significant.

## Results

**FIGURE 1:**
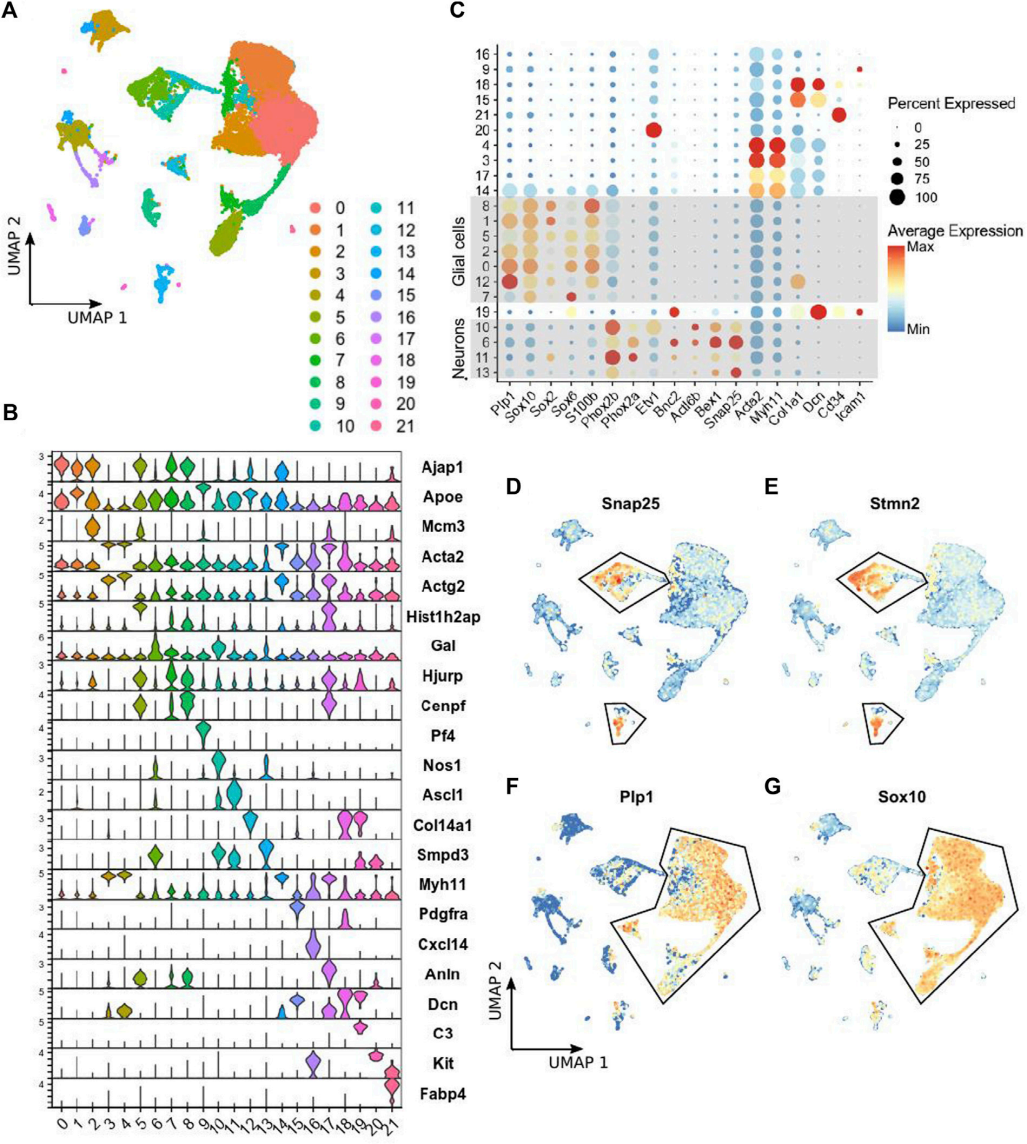
scRNA-seq of LMMP ENS cells from *Ednrb* ^*−/−*^ mice. **(A)** UMAP projection showing 22 clusters of cells identified by applying scRNA-seq to tdTomato-positive cells sorted from the LMMP of *Ednrb* ^*−/−*^ mice. **(B)** Violin plot showing expression of the top marker gene identified for each cluster identified in **(A)**. **(C)** Dot plot showing the expression of the indicated genes, including selected glial and neuronal marker genes, in each cluster of cells identified in **(A)**. Dot size indicates the percentage of cells in each cluster with >0 transcripts detected, color indicates the mean level of expression relative to other clusters. **(D,E)** UMAP projections showing expression levels of the indicated neuronal marker genes in single cells. Outlined cells were considered to be neurons and used for comparison to WT mice. The scalebar for gene expression is the same as in **(C)**. **(F,G)** UMAP projections showing expression levels of the indicated glial marker genes in single cells. Outlined cells were considered to be glial cells. The scale bar for gene expression is the same as in **(C)**.

**FIGURE 2:**
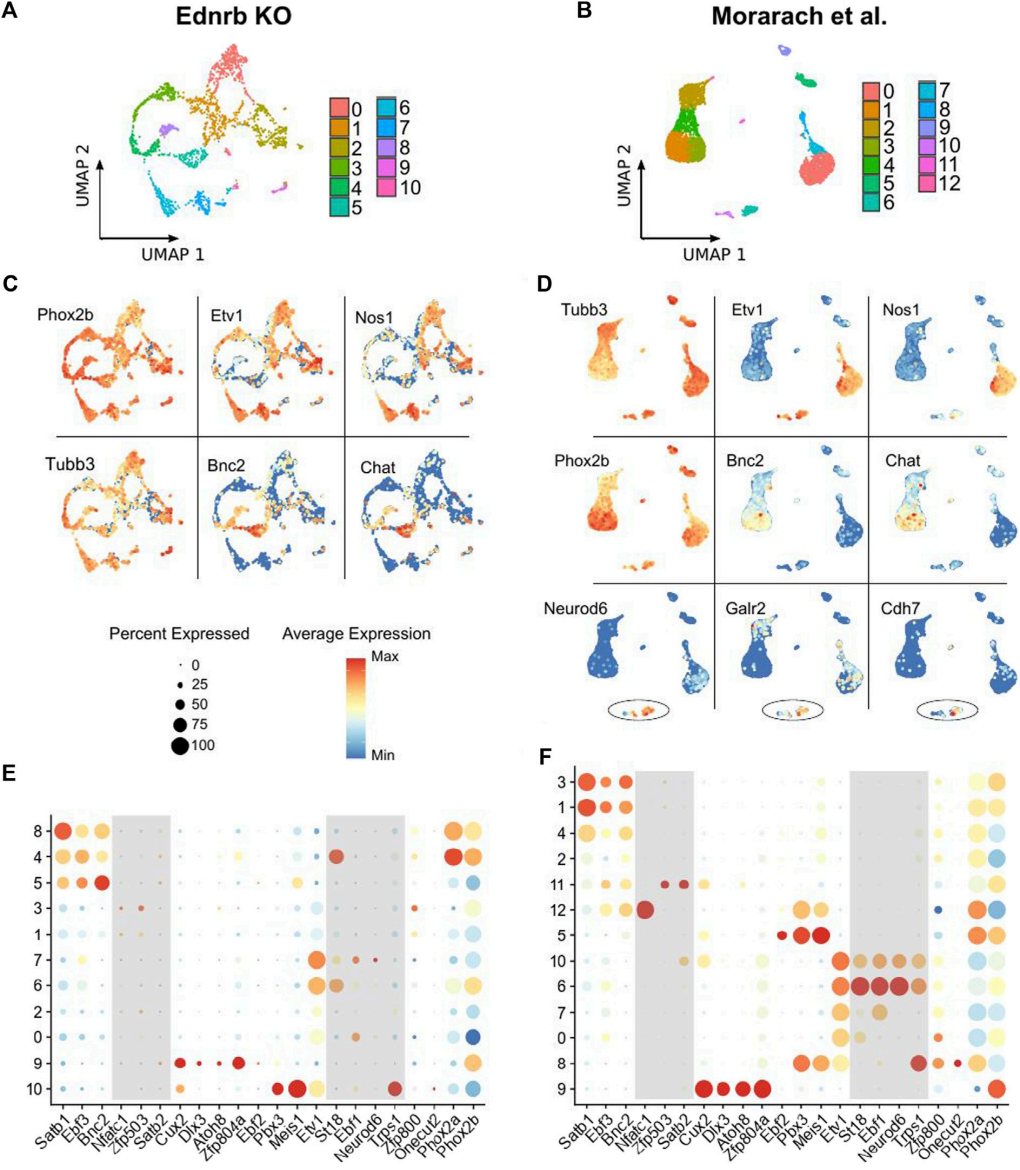
Comparison of scRNA-seq results from *Ednrb* ^*−/−*^ mice and WT mice. **(A,B)** UMAP projection showing neuronal clusters identified among the neurons in the *Ednrb* ^*−/−*^ and WT scRNA-seq datasets. **(C,D)** UMAP projections showing expression of the indicated genes in the *Ednrb* ^*−/−*^ and WT scRNA-seq datasets. **(E,F)** Dot plots showing expression levels of the indicated glial marker genes in either the *Ednrb* ^*−/−*^ or WT scRNA-seq data. Dot size indicates the percentage of cells in each cluster with >0 transcripts detected, color indicates the mean level of expression relative to other clusters. Shaded boxes denote groups of genes that mark distinct populations in the WT data but not in the *Ednrb* ^*−/−*^ data.

ScRNA-seq was performed on the small intestinal myenteric neurons and glia isolated from P14 *Ednrb* ^*−/−*^ mice expressing a tdTomato reporter in all neural crest-derived cells. In addition to ENS cells, we captured numerous populations of mesenchymal and hematopoietic cells (Figures 1A,B). The majority of cells were enteric glial cells marked by *Plp1*, *Sox10*, *Sox2*, *Sox6*, and *S100b* expression, and neurons marked by genes such as *Phox2b*, *Tubb3*, *Actl6b*, and *Snap25* (Figures 1C,D). We focused our analysis on clusters 6, 10, 11, and 13, which include cells expressing these canonical neuronal marker genes. For comparison, we utilized a published dataset of enteric neurons from P21 wild-type (WT) mice (Morarach et al., 2021). Analysis of this published data yielded similar results to what was previously reported, including distinct groups of neurons marked by *Etv1* and *Bnc2* transcripts, nitrergic neurons marked by *Nos1,* and cholinergic neurons expressing *Chat* (Figures 2B,D).

After removing all non-neuronal cells from our *Ednrb* ^*−/−*^ dataset, we again performed dimensional reduction and clustering on the 2060 neurons that remained. We identified 11 clusters of enteric neurons (Figure 2A), which is slightly fewer than the 13 clusters in the WT dataset (Figure 2B). Similar to the WT neurons, our *Ednrb* ^*−/−*^ neurons expressed the pan-neuronal markers *Tubb3* and *Phox2b*, and non-overlapping groups of *Etv1*-expressing and *Bnc2*-expressing neurons were seen (Figures 2C,E). We also found clusters of cells expressing both *Nos1* and *Chat*, indicating that we isolated both nitrergic and cholinergic neurons, respectively, and confirming successful isolation and sequencing of a diverse group of enteric neurons.

The smaller number of clusters identified in our dataset versus the WT dataset raised the possibility of absent neuronal subpopulations within the ganglionated SI of *Ednrb* ^*−/−*^ mice. To investigate this, we evaluated expression of a set of transcription factor genes previously shown to mark enteric neuron populations (Morarach et al., 2021). Within the WT dataset, clusters of neurons were marked by all the combinations of transcription factor genes reported by Morarach et al. (2021; Figure 2F). However, in our *Ednrb* ^*−/−*^ dataset, we observed a paucity of cells marked by certain transcription factor combinations. Specifically, there was a lack of the neuronal cluster marked by high expression of *St18*, *Ebf1*, *Neurod6*, and *Trps1* (Figure 2E), which was reported to label a population of neurons that express the *Gad2* gene. This gene encodes the glutamate decarboxylase two enzyme, which catalyzes the production of *γ*-Aminobutyric acid (GABA) (Karlsen et al., 1991; Morarach et al., 2021).

**FIGURE 3:**
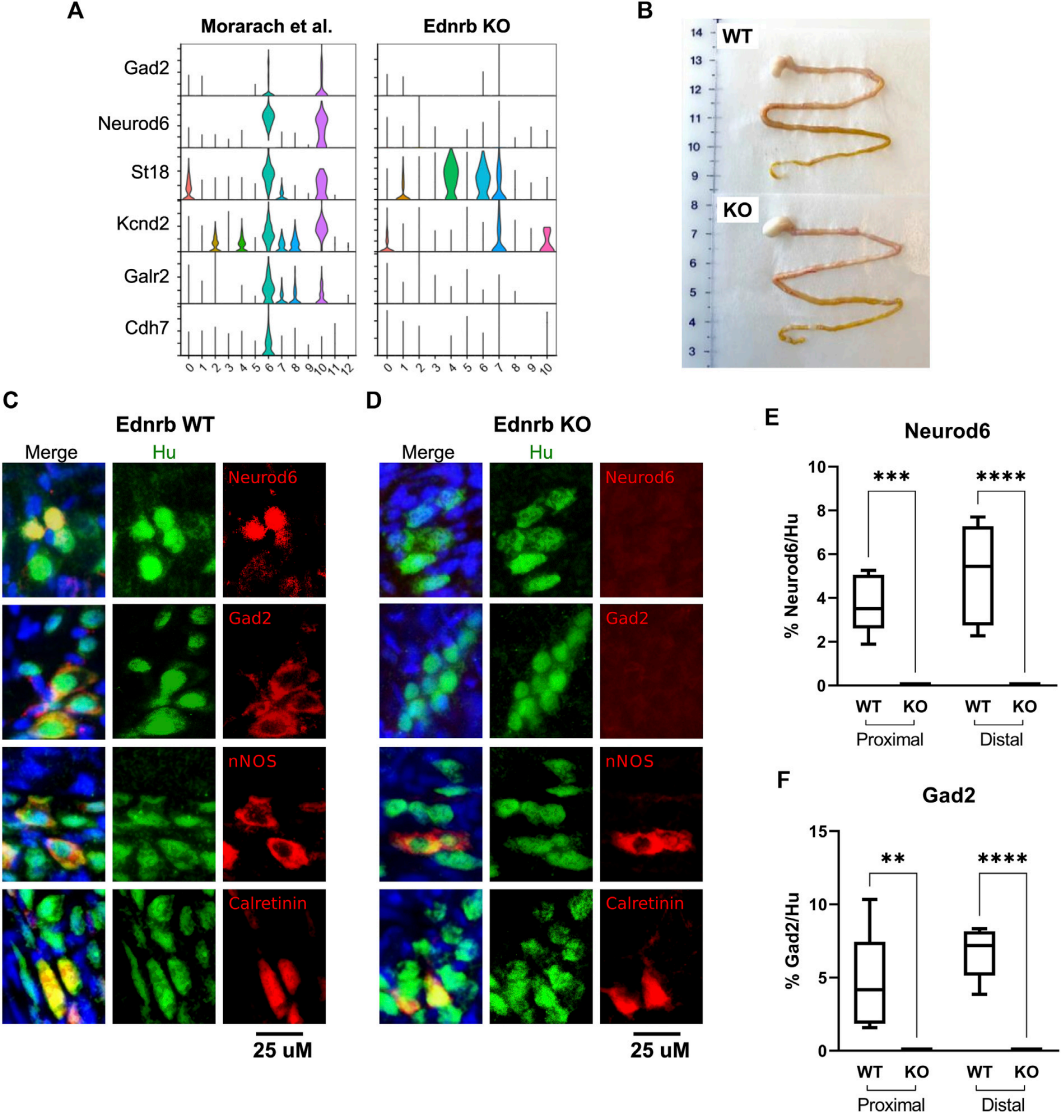
*Ednrb* ^*−/−*^ mice lack a GABA-ergic neuron population marked by Neurod6 and Gad2 in the SI of WT mice. **(A)** Violin plots showing expression of the denoted GABA-ergic neuron marker genes in WT and *Ednrb* ^*−/−*^ SI. **(B)** Gross appearance of the stomach and SI of WT and *Ednrb* ^*−/−*^ mice. **(C)**, **(D)** High-power microscopy images showing immunostaining for the denoted proteins in the SI of WT and *Ednrb* ^*−/−*^ mice, as indicated. Merge images include DAPI (blue). **(E,F)** Quantification of the proportion of Hu + neurons in each ganglia that are immunoreactive for Neurod6 or Gad2, as indicated. Box-and-whisker plots indicate mean (horizontal bar), interquartile range (boxes), and range (whiskers). ** indicates *p* < 0.01, *** indicates *p* < 0.001, **** indicates *p* < 0.0001.

We examined expression of other reported markers of this cluster, including *Gad2*, *Kcnd2*, *Galr2*, and *Cdh7*, in each dataset. While two clusters were marked by these genes in the WT dataset, there was a striking absence of neurons marked by this combination of transcripts in our *Ednrb* ^*−/−*^ data (Figure 3A). The original publication by Morarach et al. identified only one cluster of neurons marked by this combination of transcripts (Morarach et al., 2021), while our re-analysis of their data identified two, which are distinguished by *Cdh7* expression (Figure 3A). It is unclear whether this is due to minor differences in data processing and clustering or reflects true biological variation not appreciated in their initial analysis. Nevertheless, these results led us to hypothesize that the SI of *Ednrb* ^*−/−*^ mice lacks this neuronal population.

To confirm whether *Ednrb* ^*−/−*^ SI lacks the neuronal subtype in question, we performed immunostaining on the LMMP layer of SI from *Ednrb* ^*−/−*^ mice and WT littermates. *Ednrb* ^*−/−*^ SI appears grossly identical to WT SI (Figure 3B). Immunostaining identified nNOS+ and Calretinin+ in both WT and *Ednrb* ^*−/−*^ animals (Figures 3C,D, and Supplementary Figure S1A, S1B), with no statistically significant differences in expression of these markers within ganglia of WT and *Ednrb* ^*−/−*^ mice (Supplementary Figure 1C, 1D). However, there was a striking absence of neurons marked by either Neurod6 or Gad2 in the SI LMMP of *Ednrb* ^*−/−*^ mice (Figures 3C,D, and Supplementary Figure 1A, 1B). We did not notice any significant differences in the proportion of neurons that were immunoreactive for either Neurod6 or Gad2 in the proximal and distal small intestine (Figures 3E,F). These data confirm our scRNA-seq findings and demonstrates that the small intestine ENS of *Ednrb* ^*−/−*^ mice lacks a specific neuronal subpopulation that is present in WT mice.

## Discussion

Post-surgical long-term GI dysfunction is commonly seen in patients with HSCR. Recent studies have demonstrated that HSCR patients experience dysmotility throughout the GI tract, including esophagus, stomach, duodenum, and colon (Miele et al., 2000). Similar observations of diffuse abnormal function throughout the GI tract have been made in mouse models of HSCR. For example, increased gastric emptying has been described in *Ednrb* ^*−/−*^ and *Sox10* ^*Dom/+*^ mouse-models of HSCR (Musser et al., 2015; Bhave et al., 2021). Complete loss of colonic migrating motor complexes has been observed in the proximal ganglionic colon of *Ednrb* ^*−/−*^, *Et-3* ^*−/−*^, and *Gdnrf* ^*+/-*^ HSCR mice (Ro et al., 2006; Roberts et al., 2008). In the small intestine, a delay in transit of carmine-red dye was reported in *Ednrb* ^*−/−*^ mice (Bhave et al., 2021). Whether the structural and morphometric ENS anomalies observed in HSCR contribute to these functional deficits proximal to the aganglionic region is still unknown.

In this study, we performed single-cell transcriptomic profiling to interrogate the composition of myenteric neurons in the small intestine of HSCR mice. This is the first study to identify an absent enteric neuronal subpopulation in the ganglionated small intestine in a mouse model of HSCR. The missing neuronal subtype is marked by the transcription factor Neurod6 and the GABA-producing enzyme Gad2 (Karlsen et al., 1991). While we have not directly shown GABA immunoreactivity in these neurons, their transcriptomic profile and immunostaining for Gad2 suggests they are GABA-ergic neurons. This same population was identified and validated previously in wild-type mice (Morarach et al., 2021), although that study also did not directly demonstrate GABA immunoreactivity. Despite this lack of direct immunostaining for GABA, no other enteric neuron populations expressing the *Gad2* gene were identified in the scRNA-seq data, making it likely that the missing neurons are a discrete population of GABA-ergic enteric neurons. GABA-ergic interneurons constitute 5–8% of myenteric neurons and are an important component of enteric neurotransmission (Krantis, 2000). Our group has previously shown that neuronal cell body density within ganglia is not altered in the small intestine of Ednrb−/− mice, although the density of ganglia within the bowel is reduced (Bhave et al., 2021). We thus speculate that other neuronal subtypes may be slightly increased within ganglia, and further studies are warranted to address this question.

If, as we postulate, the missing neurons are GABA-ergic, the effects on motility are likely to be complex.. GABA is known to have both excitatory and inhibitory effects on GI motility depending on its specific activation of ionotropic GABA_A_ vs. metabotropic GABA_B_ receptor (Grider and Makhlouf, 1992). For example, a GABA_B_ agonist, baclofen, increased duodenal cyclic motility in rats, while a GABA_A_ agonist, muscimol, was found to have an inhibitory effect on the peristaltic reflex (Fargeas et al., 1988). Furthermore, GABA_A_ and GABA_B_ receptors are differentially expressed depending on the region of the GI tract and the species being studied (Auteri et al., 2015). We speculate that the loss of GABA-ergic neurotransmission represents a potential mechanism to account for the GI dysmotility observed in the proximal ganglionated bowel of the *Ednrb* ^*−/−*^ model of HSCR, and possibly also the *Sox10* ^*Dom/+*^ model (Musser et al., 2015; Bhave et al., 2021). Future experiments are needed to rigorously test this hypothesis. Whether an analogous population of neurons is also absent in the ganglionated bowel of human HSCR patients remains to be determined.

Our findings demonstrate the value of using high-quality, published scRNA-seq data as a point of comparison for disease models. While control data produced under the same conditions in the same laboratory as the disease model remains the ideal standard, the cost and time associated with large-scale scRNA-seq studies can make this impractical for many projects. The recent proliferation of publicly available ENS scRNA-seq data will undoubtedly offer the ENS research community an invaluable resource (Guyer et al., 2022).

## Data Availability

The datasets presented in this study can be found in online repositories. The names of the repository/repositories and accession number(s) can be found below: https://www.ncbi.nlm.nih.gov/, GSE199683.

## Supplementary material

Supplementary Figure S1 Low-power images of ganglia and quantification of Nos1 and calretinin immunostaining. **(A,B)** Low-power microscopy images showing immunostaining for the denoted proteins in the SI of WT and Ednrb-/-mice, as indicated. Merge images include DAPI (blue). **(C,D)** Quantification of the proportion of Hu+ neurons in each ganglia that are immunoreactive for Nos1 or calretinin, as indicated. p > 0.05 for all.
