# Supplementary figures and images for: Ednrb −/− mice with hirschsprung disease are missing Gad2-expressing enteric neurons in the ganglionated small intestine
Source: Front Cell Dev Biol. 2022 Jul 26;10:917243. doi: 10.3389/fcell.2022.917243 (PMC9360620; doi:10.3389/fcell.2022.917243)

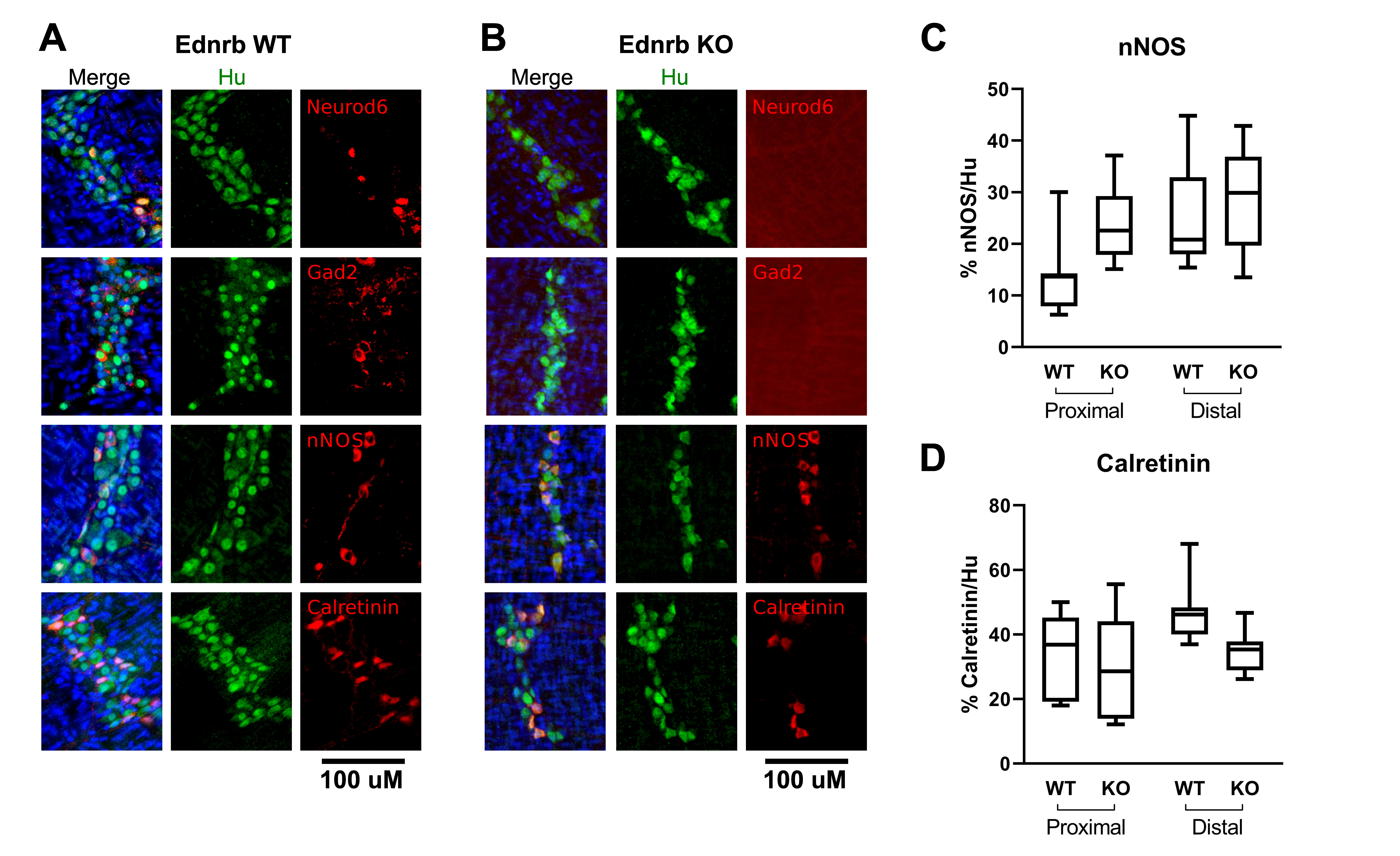

Supplement: Supplementary file 1 [file Image1.JPEG]
